# Enhancing medical students’ reflectivity in mentoring groups for professional development – a qualitative analysis

**DOI:** 10.1186/s12909-017-0951-y

**Published:** 2017-07-14

**Authors:** Gabriele Lutz, Nina Pankoke, Hadass Goldblatt, Marzellus Hofmann, Michaela Zupanic

**Affiliations:** 1 Integrated Curriculum for Anthroposophic Medicine (ICURAM), Chair for Medical Theory, Integrative and Anthroposophic Medicine, Department for Health, Faculty of Medicine, Witten / Herdecke University, Gerhard Kienle Weg 4, 58313 Herdecke, Nordrhein-Westfalen Germany; 2 Department of Psychosomatic Medicine, Gemeinschaftskrankenhaus Herdecke, Herdecke, Germany; 3 Witten / Herdecke University, Witten, Germany; 4 Department of Nursing, University of Haifa, Haifa, Israel; 5 Office for Student Affairs, Department for Health, Faculty of Medicine, Witten / Herdecke University, Witten, Germany

**Keywords:** Communication, Medical education, Reflective practice, Mentoring, Curriculum development

## Abstract

**Background:**

Professional competence is important in delivering high quality patient care, and it can be enhanced by reflection and reflective discourse e.g. in mentoring groups. However, students are often reluctant though to engage in this discourse. A group mentoring program involving all preclinical students as well as faculty members and co-mentoring clinical students was initiated at Witten-Herdecke University. This study explores both the attitudes of those students towards such a program and factors that might hinder or enhance how students engage in reflective discourse.

**Methods:**

A qualitative design was applied using semi-structured focus group interviews with preclinical students and semi-structured individual interviews with mentors and co-mentors. The interview data were analyzed using thematic content analysis.

**Results:**

Students’ attitudes towards reflective discourse on professional challenges were diverse. Some students valued the new program and named positive outcomes regarding several features of professional development. Enriching experiences were described. Others expressed aversive attitudes. Three reasons for these were given: unclear goals and benefits, interpersonal problems within the groups hindering development and intrapersonal issues such as insecurity and traditional views of medical education. Participants mentioned several program setup factors that could enhance how students engage in such groups: explaining the program thoroughly, setting expectations and integrating the reflective discourse in a meaningful way into the curriculum, obliging participation without coercion, developing a sense of security, trust and interest in each other within the groups, randomizing group composition and facilitating group moderators as positive peer and faculty role models and as learning group members.

**Conclusions:**

A well-designed and empathetic setup of group mentoring programs can help raise openness towards engaging in meaningful reflective discourse. Reflection on and communication of professional challenges can, in turn, improve professional development, which is essential for high quality patient care.

**Electronic supplementary material:**

The online version of this article (doi:10.1186/s12909-017-0951-y) contains supplementary material, which is available to authorized users.

## Background

### Professionalism now and in the future

Knowledge, skills and professionalism are pivotal elements in expert patient care and the relevance of professionalism and professional competence has received increasing attention in medical education in the last decades [1, 2]. Without this competence, the quality of patient care suffers [3, 4]. However, despite the increase in research on the topic, professional competence has remained hard to define and has differing definitions [5–7]. In line with Hodges et al. (2010), we define it as the knowledge, skills and attitudes necessary to handle individual work or study-related intrapersonal, interpersonal or institutional and societal challenges in the best possible way [8].

There are many professionally competent physicians, but there is also significant room for improvement [5]. For example, students encounter poor role models in their clinical studies [9, 10] or clinical teachers do not feel prepared to take on the role of supervisors in professional competencies [3]. And physicians’ professional competence, or rather the lack of it, is high on the patients’ complaints lists [11]. Furthermore, although professional competence is seen as important in the process of educating medical doctors in the best possible way, it still seems to decline during medical education [9, 12, 13].

In the future, digitalization will probably place increasing demands on professional competence, and, to improve future curricula, international educational research has identified the need to develop educational programs that include professional skills such as creativity, critical thinking, learning to learn, metacognition, citizenship and communication and collaboration [14, 15].

### What we know about the teaching of professionalism

Not only is there a lack of consensus on how to define professional competence but the best ways of teaching and assessing it also remain a matter for further research [5]. In recent decades, efforts to standardize the assessment of teaching have led to more standardized learning, allowing assessment goals to be met [14]. However, professional development is a process that can only partly be standardized and taught in courses [5, 16]. It is mostly a personal process in which learning is mediated by the social environment [17]. In this process, knowledge, skills and attitudes, including values, have to be personally aligned with the rules and values of others, such as patients, colleagues, teachers, teams, institutions and society, in a flexible and, for the individual, satisfying way. This alignment process occurs, at least in parts, outside the individual’s awareness through passive adaptation to hidden curricula, which are implicitly provided through the environment [18]. Unfortunately, these hidden curricula do not always serve as ideal models for developing good professional conduct, and they differ from contents of professionalism courses [19]. Furthermore, research has shown that students need support in their professional development if they are to avoid the erosion of professionalism and the development of attitudes such as othering, distance, detachment and dehumanization [20, 21].

Professional development is an ongoing, lifelong process [22]. Practicing it early and nurturing it longitudinally and throughout institutions, focusing on transformational phases in real settings appear to be important [5, 23]. Longitudinal and integrated curricula have been shown to improve psychosocial skills and humanistic attitudes, even when evaluated 10 years after medical school [24, 25]. However, professionalism is mostly taught in separate courses, often paper-case-based [5].

The two major elements of transformative learning in professional development are critical reflection and participating in dialectical or reflective discourse with others, thus allowing the best reflective judgement to be validated [6]. These elements are most effective in the learning process when they are based on authentic experiences in real settings with positive role models e.g. in mentoring [5, 6, 26, 27].

Reflection on an action can be repeatedly trained in reflective practice interventions, such as mentoring, thus allowing reflexivity to be developed. According to Archer (2007), reflexivity is a continual internal dialogue using language, including emotions, sensations and images, where people engage in twofold positions, mediating between their own concerns and the social contexts [28]. Through this process, an understanding of self and others can be deepened, intra- and interpersonal competence improved and thus professional development enhanced [29]. In addition, transformative learning calls for communicative learning in a trusting, social context, in other words, for reflective discourse [30]. Mezirow (2003) considers reflective discourse to involve assessing beliefs, feelings and values with others [31]. It is seen as the key ingredient in the process of transformational learning [32].

Reflection and reflective discourse usually start with the recognition of a so-called disorienting dilemma, which is a professional challenge or misfit. Following this recognition, an individual’s mental frames or premises and the environment have to be actively reflected upon and options for possible actions explored in order to develop autonomous and personally satisfying solutions. Through this process, satisfying professional roles can be developed and, at the same time, the flexibility to adapt to changing environments can be enhanced [33, 34]. Furthermore, in mentoring, reflection and reflective discourse positively affect not only the mentees but also the mentors in that energizing and gratifying experiences occur, a win-win situation referred to as generativity [35].

Further research has provided interesting results on individual aspects of professionalism and mentoring. While there is general consensus that professionalism should be part of medical curricula, the specifics about the timing, the integration and the setup of successful curricular elements are still unclear [6]. Peers seem to be especially important in the transmission of professional values and seem to increase participation rates in mentoring programs [36]. Although there are many positive outcomes of reflection, it is not regarded highly by all students [37] and only a minority participate in mentoring programs, mostly the high performers [38].

In an attempt to take into account all these aspects into curricula planning, a mentoring program for reflection on and discussion of professional study issues was initiated for first-year medical students at Witten/Herdecke University (WHU). In this program, small groups of students (mentees) meet with faculty (mentors) and more experienced peers (co-mentors) to reflect on and discuss personal professional topics they encounter in their study environment, but also on what it means to become a physician. Topics can be ethical dilemmas encountered in their early practice rotations in general practice, conflicts in study-groups, stress before exams etc. The mentoring tandems, each consisting of a mentor and a co-mentor who volunteer, are dedicated teachers with high professional standards who know the institution from different perspectives.

## Methods

### Aim

This study aimed to advance understanding of whether reflective discourse on professional challenges in groups at the beginning of medical school is regarded as useful for professional development and which factors hinder or enhance how students engage in the groups.

### Setting

In recent years, faculty have reported that student attitude and behavior have been changing and first-year students have been asking for more support regarding personal professional development. Thus, a mentoring program enrolling all preclinical students at the WHU in Germany was initiated in 2013. The WHU has a longstanding focus on personal professional development, integrating factors such as personal student selection, student participation within and outside the medical school, mandatory courses in humanities and early practical involvement in clinical care [39]. During the clinical part of the curriculum, some of the ward rotations are accompanied by clinical reflection training, in which the students reflect on experiences they encountered on the wards [40].

All preclinical students were divided up into mentoring groups of eight to nine members. Attendance in the groups was not compulsory, but students were asked to participate at least during their first semester. The group format was chosen because physicians usually work in teams and therefore need to be able to reflect and communicate in groups. A randomized composition was used for setting up the groups, in line with team compositions in medicine.

The mentors were selected according to their teaching reputation: Dedicated clinical (outpatient and inpatient) and preclinical teachers with high professional standards, who could serve as good role models, were asked to be voluntary members in the program. Co-mentors were recruited on a voluntary basis. In a 90-min workshop, the mentors and co-mentors were trained regarding competency-based education and the role of the professional within this concept. They received information about the university’s educational goal to educate students so that they become lifelong learning, reflective physicians able to handle individual situations responsibly and in the best possible way. The methods used in the groups such as reflection and discussion of current questions, problems, challenges and how to cope with frequently encountered problems were described and discussed. Furthermore, the setting, practical recommendations and group rules were provided.

The goal of the new program is to support personal professional development through reflection on and discussion of individual, work-related challenges and dilemmas.

### Sample

To obtain a differentiated, heterogeneous picture of the program, all group members were asked to participate in interviews. In 2014, the mentees, mentors and co-mentors were contacted personally during the semester introduction and subsequently via e-mail. Three follow-up reminders were sent, in line with the Dillman method [41].

In Germany, studies in medical education do not require ethical approval [42]. Nevertheless, the welfare and protection of the participants were guaranteed by respecting their rights, privacy and dignity. The participants all took part in the interviews voluntarily and agreed to the publishing of the anonymized data. All personal identifiers have been removed from the data.

### Data collection

The students were interviewed in focus groups. We used focus groups because we wanted to induce a discussion among the students, which could lead to a wider range of perceptions, insights and ideas. In groups, comments by one person can trigger additional responses from others [43]. Individual interviews were conducted with the mentors and co-mentor since their time constraints did not allow group arrangements. The interviews were conducted in German by a trained interviewer (NP) using an interview guide (Additional file 1).

The interview guide was developed by the authors based on pilot evaluation data. These were retrieved from questionnaires given to participants at the beginning of the first group session. The questions were related to their expectations and concerns. Information was also gained verbally in informal evaluation rounds after the first mentoring semester. These pilot data were used to shape the interview guide questions.

Focus group interviews lasted between 40 and 60 min and individual interviews between 20 and 35 min. Participants were asked about experiences regarding the reflection groups; special focus was placed on helpful and hindering experiences regarding their current study situation but also on expected effects on their professional development. Questions were also asked about the group format and the early introduction of the program.

All interviews were audio-recorded and transcribed verbatim. The German citations used in this manuscript were translated by a professional native English-speaking translator. To ensure anonymity, only the participants’ group affiliations and not their personal identifiers, including age or sex, are mentioned in the citations.

### Study design

Since the educational intervention was developed to support innovation development in a complex environment, the format of a developmental evaluation, as defined by Patton (2011), was considered an appropriate design for this study [44]. The concept of Patton’s approach is to involve members of a development team, who collaborate to conceptualize, design and test new approaches in a long-term, ongoing process of continuous development. In this study, three authors were involved in developing the program: GL, a clinician and teacher in communication, professionalism and psychosomatic medicine; MH, the student dean of the faculty; MZ, a psychologist working in the student deanery. The interviews were conducted by NP, a psychology student and social worker, who was not involved in the development of the program. She was also involved in data analysis. HG was involved in interpreting the data and in drafting the manuscript. MH and GL were involved as group mentors; NP, HG and MZ were not.

The goal of the interviews was to gain a deeper understanding of factors influencing the willingness to engage in the reflective groups, such as inner beliefs, experienced stories, emotions and attitudes.

### Data analysis

Data from the interviews were analyzed in three steps: First, two investigators (GL, NP) read each transcript and independently coded them using thematic content analysis [45]. Second, the codes were, again independently, grouped into categories using the qualitative data analysis software MAXQDA. These categories, involving primary and secondary themes, were discussed among the coders and refined, producing a preliminary framework of categories.

In a third step, with the preliminary category framework in mind, the author MZ, who was not actively involved in the first step of the analysis, read the transcripts, looking especially for factors that were contradictory or overseen. Again, discrepancies and new perspectives were discussed and the categories refined to the final results.

## Results

Of the 168 contacted students, 14 consented to participating in the focus group interviews. Eight mentors and one co-mentor agreed to participate in individual interviews. In a first review of the data, we had the impression that content saturation was reached. The demographics of participants are given in Tables 1 and 2.

**Table 1:** Characteristics of interview participants (mentees, focus groups)

Participants, n	14 (4 groups of 3, 1 group of 2)
Focus groups, n	5
Academic year	1
Age, years (mean, range)	(22, 20–26)
Male/female	6/8

**Table 2:** Characteristics of interview participants (mentors, co-mentors, individual interviews)

Participants, n	9
Mentor/Co-mentor (year)	8/1 (5)
Discipline	6 physicians:
	4 general medicine
	1 child psychiatry
	1 psychosomatic medicine
	1 student deanery
	1 deanery
Male/female	3/6
Age, years (mean, range)	49 (31–61)

The interviews revealed both positive and negative attitudes regarding the reflection on and communication of intra- and interpersonal professional topics early in medical school.

### Medical students’ positive attitudes of reflective group work

Students’ attitudes regarding the usefulness of reflecting real, study-related intra- and, interpersonal topics at medical school were diverse. For instance, a student described how the group split early on.

*“Well, I had, for example, the impression that the group split very early on. And that many then no longer came, but that then those who remained, they wanted to do that and they could use it.”* (Mentee).

Some mentees expressed positive attitudes towards the program implementation. They described intra- and interpersonal skills and attitudes as essential in clinical practice and stated that communication and reflection to enhance them should be practiced early on. Mentors stated that they felt gratified to be able to watch young people develop in this process.

*“I somehow viewed that this program was something that is a sort of attempt, well, to develop the personality, […] and because, well, how one develops one’s personality, […] I think that, well one does that perhaps through an exchange with people with different views, with other life styles, and I consider that really still to be part of my studies.”* (Mentee).

*“And I notice that that is a really big present for me, that I am able here to see how ten young people begin to develop their lives.”* (Mentor).

In many interviews, the notion was expressed that groups seemed to become successful, to really engage into meaningful reflection and communication, when they dared to address personally challenging situations. The moment when participants seem to be able to find the courage and open up for personally meaningful topics was described as a pivotal point in starting a meaningful reflective group process and learning professionally.

*“And then, however, there was the moment when one person was brave enough. OK, now I’ll simply say something and say something that really, perhaps it is now a bit more intimate, but then that’s how it now is. And then suddenly there was a discussion and all of a sudden the people were talking to one another. And that, well, that one also, when one doesn’t say anything intimate, then nothing happens. Well, I think, why not, […] And yes, I find that, well, that was really an interesting moment, how something can change like that, an atmosphere in a room.”* (Mentee).

A precondition for this pivotal point was seen in the development of an atmosphere of the group flourishing as a group, an atmosphere of security and interest in each other, which allows the individuals to open up and speak about personally challenging topics.

*“I think that is almost a prerequisite, that one can also perhaps express one’s fears or something like that, that one feels good. And I noticed, when I think back to the first meeting: I don't know how he managed that, but right from the beginning it was very much as if, so that one certainly felt that one could somehow say something. […] That is, I think, also very important because then one feels safer saying something.”* (Mentee).

Two conditions had to be fulfilled for a meaningful reflective group process to take place: the group had to be successful in developing bonding, trust and interest and the students had to be courageous and dare to openly address meaningful challenges. Students felt that they could learn from reflecting and talking about the problems and, through this process, flourish as individuals and improve their understanding of themselves and others, and thus increase intra- and interpersonal professional competencies. They felt they could talk more openly and actively about problems; they also seemed to become able to use feedback from others, gain more perspectives on discussed situations, be more open towards people who were different and become more reflective.

*“Through that, to reflect on one’s own behavior, that I learn how I behave, how I appear to others, how I shape things, when I think about that or reflect on it for the first time or learn […] at least I have the feeling that one can then somehow learn that; perhaps because there is someone else in the group that already does that really well, or perhaps has developed a strategy how he thinks about things or perhaps only has an approach to reflecting on things.”* (Mentee).

**Fig. 1:**
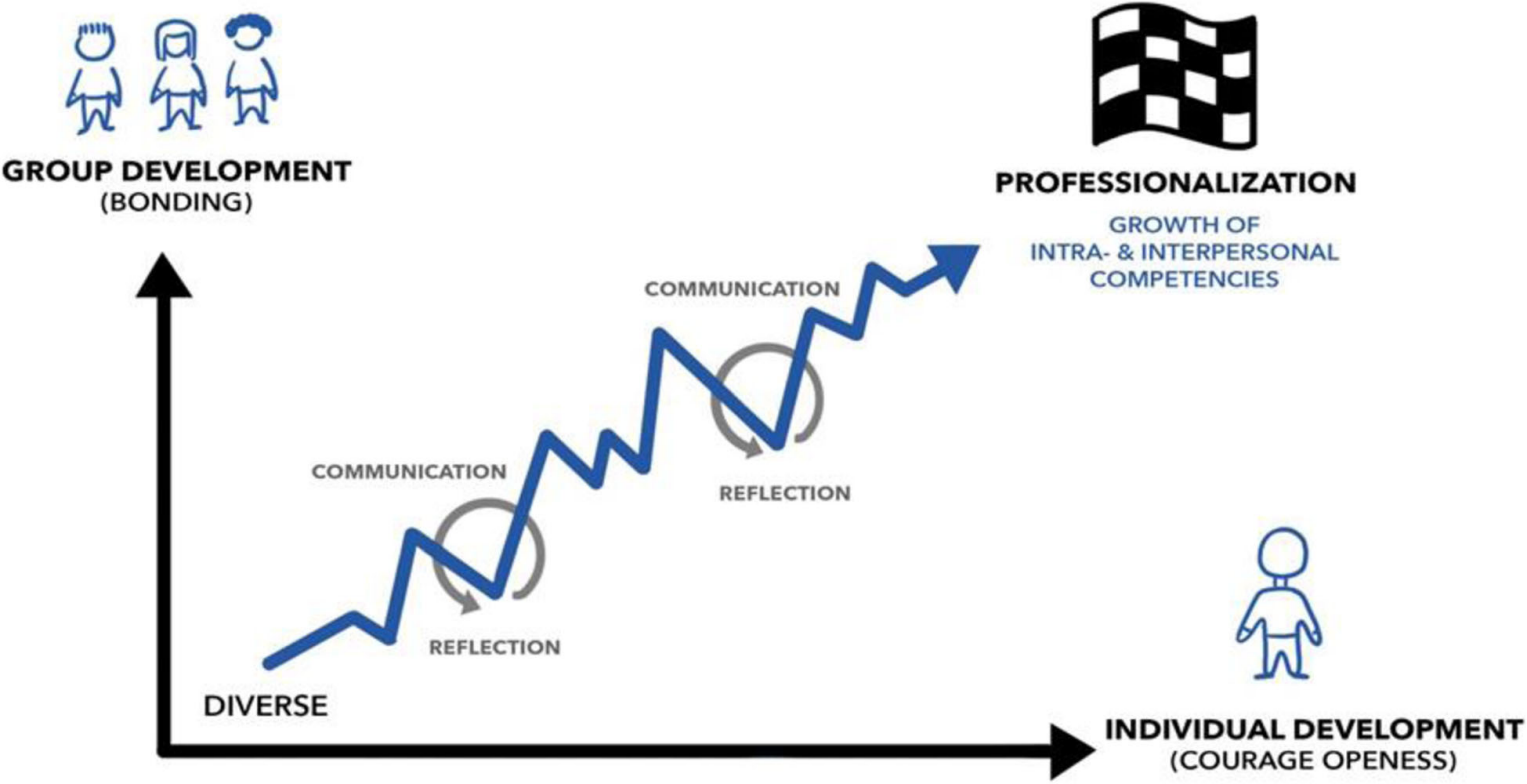
Positive attitudes towards reflective group work

Figure 1 shows how students’ positive attitudes towards reflective group work develop if the group succeeds and can thus, through this process, engage in reflective discourse on meaningful challenges to developing professional competence.

### Medical students’ negative attitudes towards reflective group work

Other students did not find these groups useful. They could not or did not want to reflect on or talk about experiences involving actual intra- or interpersonal issues. Personal aversive attitudes towards participation in the groups had different sources. Three relevant categories were articulated: factors related to the framing and setting up of the program, negative interpersonal attitudes and behaviors, and negative intrapersonal attitudes.

Several statements related to the program’s framing and setup. Some students seemed not to see any benefit from the group work for their professional development or why or how improving their performance as physicians was related to practicing their abilities to reflect on and communicate personal professional issues. It seems that the goals, content and relatedness to the rest of the curriculum and to the tasks later in professional life as physicians were not clear to them.

*“I found […] that somehow no-one really knew at the beginning, I don’t know, I think the co-mentor and the mentor also didn’t really know at the beginning where it was all going to.”* (Mentee).

The students also named different hindering interpersonal behaviors that impeded openness, trust and interest within the group, such as lack of reliability, breaking confidentiality rules, disrespect. It was also stated that group members did not fit personally to one another, and therefore they could not trust and open up to each other and subsequently no meaningful learning experiences could occur.

*“And they then, well, said again that, during the semester, [being] social simply isn’t that easy, and that they wouldn’t trust themselves to talk about difficulties in front of certain people, that they wouldn’t feel protected there and that it would be difficult.” (*Mentor*).*

Some students revealed negative intrapersonal attitudes towards such groups. Some expressed concerns about strongly emotionalized discussions in such groups. Others stated that they did not want to personally expose themselves to unfamiliar students and faculty in a university setting. This attitude seemed partly to be rooted in the traditional view of medical education as the acquisition of knowledge and skills, and the students felt that personal or interpersonal issues did not belong in educational groups. These issues were perceived more as something to be discussed outside, with friends and trusted people, or later in medical education. All in all, these participants did not want or dare to open up for group reflection.

*“[…] at least I think that, at the moment, I don’t see that this entire program is of much use to me because the problems I have, I can discuss those better in a small group. And can talk about them better with people who know me well.”* (Mentee).

**Fig. 2:**
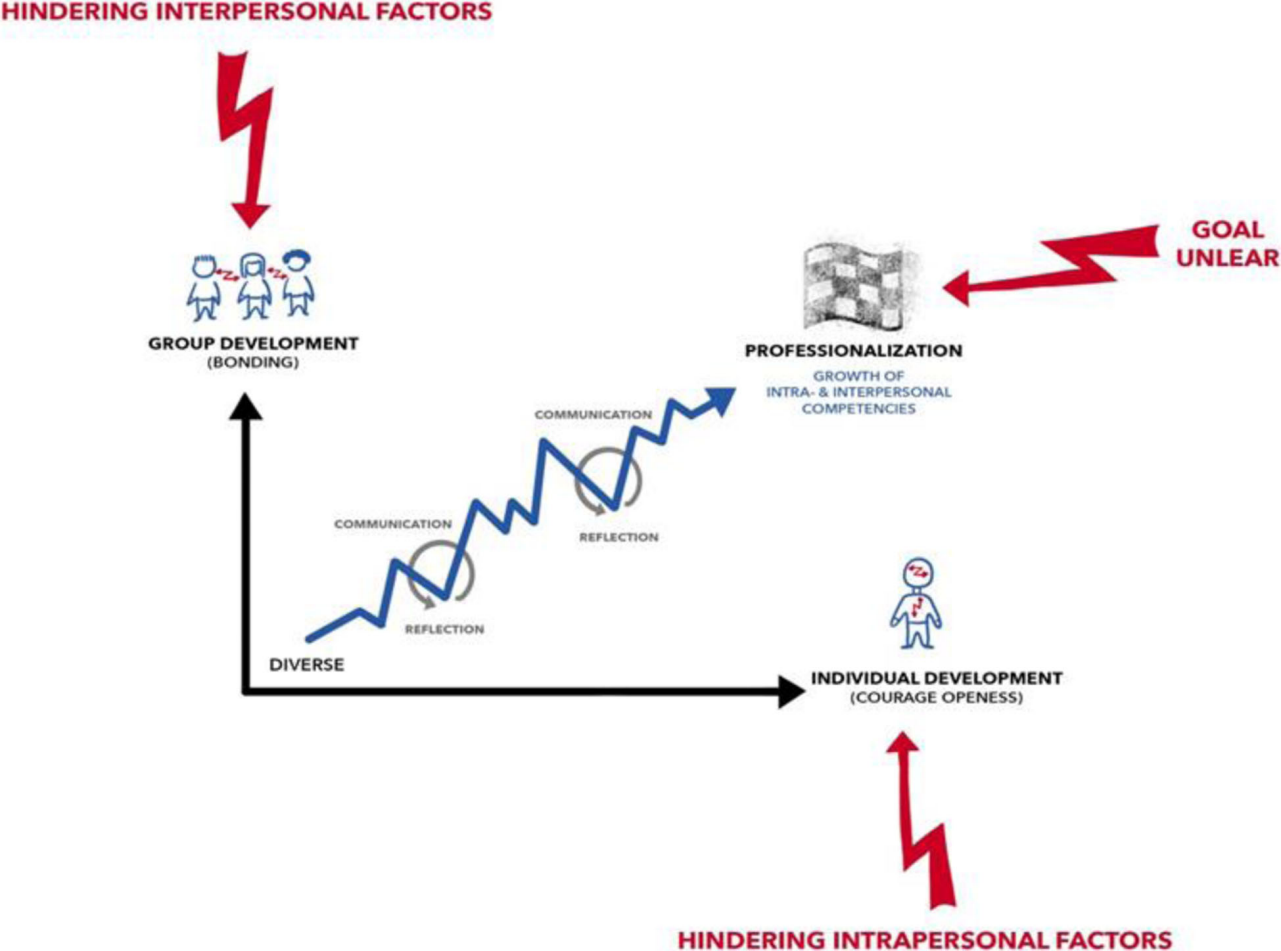
Factors impeding the motivation to engage in reflective discourse in groups

Figure 2 gives an overview of different factors that impede the motivation to engage in reflective discourse in groups.

### Curricular factors enhancing reflection in mentoring groups for professional development

The participants’ statements allow the identification of setup factors that might possibly be helpful in developing successful group work.

Explaining thoroughly why professional reflection and discourse is important and explaining curricular expectations was regarded as helpful. In addition, meaningfully integrating the group work into the whole curriculum was named as essential in experiencing the relevance of reflection. Linking the group encounter to experiences that can evoke dilemmas were described, e.g. group dynamics in study groups, difficult encounters in the general medicine rotations, preparation for the first major exam.

*“That I would simply find it interesting to have the meetings... perhaps naturally right at the beginning, of course, because there the first impressions are so crass, after the first general practitioners’ internship and then perhaps after the exams or something like that, […] or somehow one also has to battle a bit with these completely new experiences or ones that are occupying one’s mind, or whatever.”* (Mentee).

The dilemma was discussed that reflection cannot be forced but that, on the other hand, without some kind of obligation students would not take part in the process. Especially the student participants stressed the need for some kind of curricular obligation to participate in the groups; otherwise, they would rather participate in learning encounters they are familiar with and they could get good test results from.

*“I don’t know whether it would be so good on a voluntary basis because I think that then many, […] wouldn’t even have taken up the offer [...] then of course no one would go or only those who, well of course one could view it positively, only those who are really interested. But perhaps not really those who could perhaps really use this help now. So, if one first persuades them and says: please just start by looking at it.”* (Mentee).

*“Well, I think that not everyone can do this. And there shouldn’t be any compulsion. But I find that a difficulty too, so it also shouldn’t really be completely voluntary.”* (Mentor).

Although not all participants fitted well into the group in which they had been placed, the mentees generally valued a randomized composition of the group with unfamiliar and diverse group members since it allowed more new perspectives to be gained and gave more mirroring possibilities.

*“And I find, the more people one comes into contact with that are not so similar to you as your friends are […] then one can, well, every individual can benefit from it because one can naturally always take something on from the right and the left, I find. Now not copy exactly, but simply in interacting. […] well it is, I believe also important because in the clinical internship one isn’t together with people, well, we, as doctors, perhaps at some point, are also not together with people who we then all want to hug.”* (Mentee).

The mentoring tandem consisting of a faculty member and a clinical student was reported as helpful. Mentees expressed the desire to have mentors and co-mentors as possible role models. Mentees did not want them to solve problems but rather wanted them to empower and be interested personally and be open regarding the mentee’s own professional development. Therefore they were regarded as facilitators, but also as group members. Both should act to help install an atmosphere of security, trust and interest, but also to encourage and support. The mentor should provide the greater professional picture, whereas older students should actively advise in current student problems.

“*Well, if at all […] the older students with the experiences related to the studies because it is really mainly about the studies of course. And the doctor had generally more, … yes that is the goal we want to aim for and listened more and brought us down to earth and basically gave us fatherly courage, like ‘you’ll manage that’*.” (Mentee).

Mentors and co-mentors also reported personal professional gain.

*“All in all, I have to say that I was very impressed by everything. Well, one always thinks beforehand: What, yet another appointment, something else. But really it was like a sort of present, to be together with the students, because we had really interesting discussions and at the end they were really open and it was easy for me and I think for the co-mentor, too. Just great to see what happened there.”* (Mentor).

**Fig. 3:**
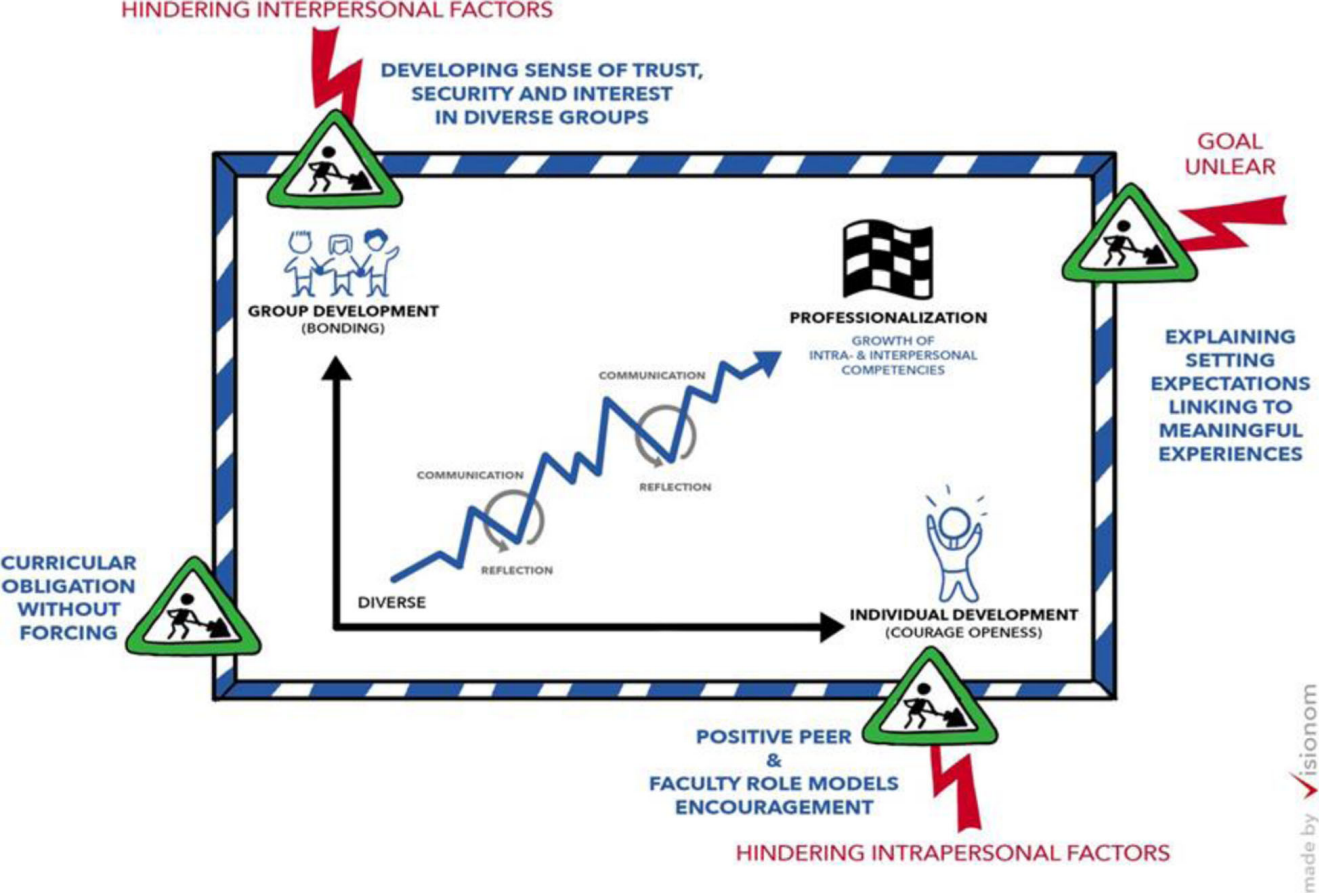
Curricular factors enhancing reflection in mentoring groups for professional development

Figure 3 shows curricular factors that might help enhance the students engaging in reflective discourse in mentoring groups for professional development.

## Discussion

Professional competencies are essential in good quality medical care [46]. Since they are developed within a social context and the clinical environment does not always provide an ideal role model, reflection groups can provide safe and trusting social contexts and learning settings in which meaningful experiences can be reflected and discussed and professional skills and attitudes developed.

Assertions made in previous studies could be confirmed through our study. The competencies needed in professional development [47–49] and later on as a physician appeared to be enhanced by the reflection groups for preclinical medical students, involving dedicated faculty and older students, where professional questions arising in the real study environment could be reflected and communicated. Students starting medical school in Germany are mostly familiar with standardized testing, teaching and learning and are not used to opening up for intra- and interpersonal aspects of education. They are used to getting very good marks for very good performance. Therefore they are unaccustomed to talking to unfamiliar others about personal and interpersonal issues and difficulties. The fact that we ask students to engage in mentoring groups can pose a disorienting dilemma [33] between premises learned in school and the new and unfamiliar environment.

Although the possible benefits of group reflection were described, mentees perceived such groups very diversely. Some students could not see any benefit from such reflection for their work as students or physicians; some encountered interpersonal problems that impeded the openness to engage. A third group seemed to have difficulties in exposing themselves personally to others because of intrapersonal mind-sets or personalities that did not allow personal reflective discourse. This group will need approaches different to reflective group discourse, ones more individually or cognitively oriented, such as one-to-one mentoring or reflective diaries. A good number of students did not want to engage initially, but they appeared to have the ability to learn if certain factors were catered for by the group. Some students seem to already have the capacity and openness to engage in reflection and reflective discourse and to assess with others their beliefs, feelings and values. They are very helpful in the groups since they can help the hesitant students to open up for reflective discourse.

The following five factors were perceived as easing the transformation of mental frames of reference to more positive attitudes towards reflection on and communication about professional intra- and interpersonal questions relating to good professional development. First, students and faculty need a comprehensive explanation of what professional competence means, how it relates to implementing reflection during different professional activities and why it is important for the delivery of high quality care. The medical school’s values should be openly stated and explained, and the reflection groups should be placed into the curriculum in a meaningful way so that students also can appreciate the benefits, e.g. a teamwork reflection at the beginning of learning groups. The importance of setting expectations regarding professional competence has been described elsewhere [5, 47, 48].

Second, since reflection and meaningful communication with others cannot be forced, mentoring programs are usually optional. Forcing students could raise resistance to engaging, especially in the current prevalent culture in medicine, where reflecting on and discussing professional problems with colleagues is rare. In our study, we expected all students to participate. It seemed that mostly those students engaged who already had some openness to personal engagement. Hesitant students tended to leave the program. Therefore students especially felt that there should be some obligation to take part in the groups. A flexible and empathic approach would seem best where students have to develop their professionalism and gain credits for it but can choose the, for them, appropriate way and possibly change groups or use individual mentors [49]. Without any obligation, they might opt to study subjects where they receive grades. This tendency is underpinned by Grant et al. (2006), who also found that students are unlikely to take up voluntary reflective learning if they do not think it relates to the curriculum and assessments [50].

Third, trust and security were perceived as the major prerequisite to opening up for reflection and discussion of meaningful challenges. If such an atmosphere can be developed, intra- and interpersonal issues inherent to these challenges can be reflected on, discussed and changed. Moreover, development is not only enhanced by the topics discussed in the group. The group process itself may even be more important in enhancing professional competency. By opening up to themselves and others, students practice gaining a better understanding of themselves and others. Therefore, first encounters should allow not only for establishing group rules but also for trust and security to grow. In his model on how to teach professionalism, Branch (2015) proposed supportive small group work as one of four major constituents besides experiential learning, critical reflection and a sufficiently longitudinal curriculum [27].

Fourth, to open up personally and emotionally in groups might be more difficult for some, but the interaction of faculty, older peers and motivated students with hesitant students can be helpful in raising the latters’ motivation to engage. Furthermore, interpersonal misfits can inhibit the group process [48]. Yet, our participants valued a diverse group composition because the more diverse the group participants, the more perspectives were generated, and the more the participants could learn. This diversity also reflects the social composition of teams later in life. The best fit cannot be foreseen in advance [49]; several attempts may be necessary to find a good fit. Overall, the mentor/co-mentor abilities seem to be more important for the success of groups than the setup of the groups [51]. Reflective discourse in diverse groups appears to be a deliberate prophylaxis of othering, and important in nurturing humanism in medicine [20, 21].

Finally, the mentoring tandems of personally interested and self-disclosing group members and facilitators were perceived positively in our study and elsewhere [26, 49, 52]. Apart from encouraging and supporting, they also had other roles. The mentor is in a better position to provide the greater professional picture, whereas older students, acting as co-mentors, are closer and more similar to the mentees and can advise in current student problems. In order to competently act as reflection guides and change agents, they were expected to reflect openly their mentees’ and their own, development, to be personally interested and to empower rather than advise in problem solving. Both, mentor and co-mentor, should act to help install an atmosphere of security, trust and interest to enable students to open up, but also to encourage and support. These findings are in line with other research [20]. In our study, and in other research, mentors and co-mentors reported personal professional gain [49, 53], as did the students, which created mutual meaning and motivation, a sense of generativity, but also a notion of connection and reconnection with teaching, core professional values and colleagues [54–56].

Successfully implementing these five factors will need commitment, time and empathetic approaches if the stakeholders for change are to be convinced. Changes on personal and institutional levels will be needed [57]. Furthermore, additional research is needed both to substantiate evidence of the impact of professional competence on outcomes and on the obstacles impeding the transmission of the existing evidence into change in practice [57, 58]. In particular, this future research could investigate issues that have arisen from some limitations to this study. First, the study only evaluated attitudes at one setting, limiting the generalizability of our findings. Since the medical school has a personal selection process in which the capability to reflect is a student evaluation factor, there may be selection bias due to its special character. Furthermore, the students who agreed to take part in the interviews may have been particularly motivated and reflective. Even though we tried to include in the interviews students with objections, they might have been more reluctant to agree to participate. Thus, the real objections to the mentoring groups might be more diverse than those captured.

In spite of these limitations, the study has been able to contribute to understanding possible avenues for improving professional competence. To the best of our knowledge, this is the first study to investigate medical students’ attitudes towards positively engaging in reflection on and communication of the professional intra- and interpersonal or institutional challenges faced during their studies. The topic could be studied from three perspectives, the mentors’, the co-mentors’ and the mentees’, thus allowing a detailed understanding of the objections to such experiences and the setup factors that might be helpful in letting students engage in the group process. These perspectives are necessary to enable as many students as possible to engage in active professional development and thus improve their intra-, interpersonal and institutional professional competence.

## Conclusions

Previous research has shown that professional competence in medical students can be enhanced by reflection on and communication of intra-and interpersonal dilemmas, as e.g. in mentoring groups. Our work confirms these results and has, additionally, identified how obstacles to introducing these processes in medical education could be overcome. Overcoming obstacles would allow more students to open up and engage in discussions and reflections that benefit their professional competence.

## Additional file

Additional file 1: Interview guide. (DOCX 18 kb)

## Abbreviation

WHU: Witten/Herdecke University
