# Supplementary material for: Enhancing medical students’ reflectivity in mentoring groups for professional development – a qualitative analysis
Source: BMC Med Educ. 2017 Jul 14;17:122. doi: 10.1186/s12909-017-0951-y (PMC5512833; doi:10.1186/s12909-017-0951-y)
Supplement: Additional file 1: — Interview guide. (DOCX 18 kb) [file 12909_2017_951_MOESM1_ESM.docx]

**Interview guide**

The following questions were used to give the interview a flexible structure. The questions aimed to cover relevant areas of the topic, but left ample room for additional material the interviewees provided.

1. I would like to talk with you about your experiences with the mentoring program in general and the experiences you made within the groups. How was this for you?
   1. What was meaningful?
   2. Can you describe any of your experiences?
2. Are there any specific memories of the group meetings?
   1. Was there anything meaningful?
   2. Was there anything surprising?
   3. Was there anything you learned?
   4. Was there anything uncomfortable?
   5. Were there any remarkable experiences? Do you have examples of pleasant or unpleasant experiences?
   6. What did you talk about? What was the method used in the meetings?
   7. What was the structure of the meetings?
3. Was there any impact of he program helpful on your professional life? To which extent was it a nuisance or useless?
   1. If it was helpful, what was it helpful for?
4. What did you see as your main task as a mentor?
5. How was the cooperation with the Co-Mentor?
   What was helpful, what was difficult?
6. What was your expectation and attitude regarding the mentoring program initially and currently?
7. Does the experience with the students in the mentoring program impact on you?

Was it interesting or could you gain or learn from it?

1. How was the group work? What was good, not so good?
2. Do you have any suggestions for improvement?
